# Differences in motor imagery strategy change behavioral outcome

**DOI:** 10.1038/s41598-022-18164-1

**Published:** 2022-08-16

**Authors:** Yuki Fukumoto, Marina Todo, Yoshibumi Bunno, Hirohisa Yoneda, Makiko Tani, Toshiaki Suzuki

**Affiliations:** 1 Physical Therapy Laboratory, Kansai University of Health Sciences, 2-11-1 Wakaba Sennangun, Kumatori, Osaka 590-0482 Japan; 2 Graduate School of Health Sciences, Graduate School of Kansai University of Health Sciences, 2-11-1 Wakaba Sennangun, Kumatori, Osaka 590-0482 Japan; 3 Graduate School of Medical Science, Kanazawa University, 5-11-80 Kotatsuno, Kanazawa, 920-0942 Japan

**Keywords:** Working memory, Motor neuron, Spinal cord

## Abstract

Kinesthetic motor imagery (KMI) involves imagining the feeling and experience of movements. We examined the effects of KMI, number visualizing, and KMI with number visualizing on the excitability of spinal motor neurons and a behavioral outcome measure in a pinch force task. Healthy participants (13 men and 8 women; mean age: 24.8 ± 5.5 years) were recruited. We compared the F-waves of the left thenar muscles after stimulating the left median nerve at the wrist during each motor imagery condition after a practice session. The KMI condition consisted of imagining muscle contraction, the number visualizing condition consisted of imagining the pinch force increasing numerically, and the KMI with number visualizing consisted of alternating between the KMI and imagining the pinch force increasing numerically. Before and after motor imagery, the time required to adjust to the target pinch force was compared. The time required to adjust the pinch force was shorter in the KMI with number visualizing condition than in the KMI and number visualizing conditions. There was no difference in the F/M amplitude ratio between each MI strategy condition, indicating the excitability of spinal motor neurons. Numerical information helped to improve the ability of participants to perform KMI.

## Introduction

Motor imagery (MI) is the mental rehearsal of a movement without performing the actual movement^1^. It has been suggested that MI and movement execution share the same neural substrates^2^. Moreover, MI and execution training have a comparable training curve^3^. Because MI and actual exercise have a similar neural basis, MI has recently been used as a rehabilitation technique. After MI, the strength of the little finger abduction was increased by 22%, but there was no change in the muscular strength in the control group^4^. Moreover, upper limb function in patients with poststroke hemiparesis was improved after a combination of physical therapy and MI^5^. In our previous study^6^, we compared precision pinch force control after MI in healthy participants. Our previous study compared the pinch force deviation from the specified target value between before and after MI. As a result, precision pinch force control was maintained in the MI group but deteriorated in the control group. These observations indicate that MI improves motor performance; however, its effect in neurorehabilitation has yielded mixed results^7^. Therefore, as described above, factors underlying the conflicting effects of MI (inhibit/improve) have not yet been clarified, and the clinical application of MI is still controversial. The conflicting effects of MI may be explained by the possibility that different MI strategies may have varying effects^8,9^.There are two types of MI: kinesthetic MI (KMI) and visual MI (VMI). KMI involves imagining the feeling and experience of a movement^10^, and primarily activates motor-associated structures, e.g., the primary motor cortex and supplementary motor area^11,12^. VMI requires self‐visualization of a movement from a first‐ (internal VMI) or third‐ (external VMI) person perspective. The first‐person perspective corresponds to the representation of a movement as if the individual takes part in the action himself, hence suggesting that he/she would visualize the movement similar to the view obtained by placing a camera on his/her head^12^.There is a strong association between KMI and motor performance outcome^13^, and the error rate of precision right finger tapping was decreased after KMI. However, VMI had a negative predictive value for performance^14^. It has been proposed that KMI is associated with feedforward modeling of a motor response, which makes it especially useful for training motor paradigms^15^. However, there are several obstacles to the implementation of KMI.

First, KMI relies on the feeling of muscle movement that can usually be achieved after training^16^. Therefore, it is important to determine the best way for participants to perform KMI correctly. KMI is difficult to execute; however, the quality of KMI can be improved by motion observation^17^ and sensory information^18^. In other words, by adding additional information to KMI, we may be able to improve the accuracy of the image. Todo et al.^8^ reported the usefulness of adding number visualizing to KMI. Todo’s report, number visualizing was defined as imagining that the pinch force increased numerically on the pinch meter display. Since VMI involves the self-visualization of a movement, we believe that number visualizing, which is not movement, is not strictly VMI. Therefore, we use the expression "number visualizing" instead of VMI in this study. Ito^19^ investigated the effect of KMI with mental activity on precision grip force control using isometric contractions. Mental activity consisted of the presence or absence of numerical information for the target value. Only KMI with number visualization improved precision grip force control. Ito's report^19^ may include the effects of the double task. However, in the same study, when the participants were asked to recite numbers backwards that were not related to the mental activity, there was no improvement in performance. Therefore, we hypothesized that high performance effects might be achieved by assigning numerical information related to the MI task to increase the success rate of KMI. Second, only a few reports have focused on spinal motor neuron excitability during KMI. In previous studies that simultaneously assessed brain activity, spinal motor neuron excitability, and precision pinch force control skills, changes in spinal motor neuron excitability, but not brain activity, were most strongly associated with precision pinch force control skills^20^. Spinal motor neuron is important as the final common pathway for motor commands, which are directly related to motor expression. Spinal motor neuron excitability can be evaluated using F-waves. An F-wave is a compound muscle action potential resulting from re-excitation (backfiring) of an antidromic impulse after distal electrical stimulation of the motor nerve fibers of the anterior horn cells^21^. However, F-wave amplitude is not constant. Therefore, F-wave amplitude must be divided by M-wave amplitude (F/M amplitude ratio). When applying electrical stimulation to a peripheral nerve, compound muscle action potentials propagate orthodromically through α-motor neurons and can be recorded from the corresponding muscles as M-waves. A constant maximum M-wave value can be recorded when using supramaximal stimulation to α-motor neurons. The F/M amplitude ratio reflects the number of backfiring anterior horn cells and the excitability of individual anterior horn cells. In other words, the F/M amplitude ratio reflects the excitability of lower motor neurons within the spinal cord. The F-wave is a waveform obtained by electrical stimulation of the nerve and cannot be obtained without electrical stimulation. In other words, electrical stimulation is not used to perform imaging, but rather to determine the changes in the spinal motor neuron excitability at rest and during each imaging task. Importantly, electrical stimulation must not interfere with MI execution. In this regard, the electrical stimulation used in the present study does not interfere with the execution of MI^22^, and we believe that electrical stimulation incorporated into the experimental system is an acceptable strategy. The activation the motor system and modulation of F-wave during MI show conflicting results. There are mixed reports that F-waves show no change^23,24^ or increase^25,26^ during MI, which needs further clarification. Bunno^9^ examined the change in spinal motor neuron excitability between rest and during KMI. The F/M amplitude ratio was higher during KMI compared to rest. Spinal motor neuron excitability is strongly associated with muscle contraction; therefore, the study by Bunno^9^ suggested that a loss of motor performance can be prevented by increasing spinal motor neuron excitability. However, this report did not provide an explanation of how spinal motor neuron excitability controls movement because it only focused on spinal motor neuron excitability. In our previous study, the excitability of spinal motor neurons was increased during MI, while precision pinch force control skills did not improve^27^. Additionally, increased excitability of spinal motor neurons during MI was not always associated with improved precision pinch force control skills. Individuals who had improved precision pinch force control skills performed vivid MI, leading to suppression of the excessive increase in spinal motor neuron excitability^28^. In addition, we hypothesized that the excitability of spinal motor neurons does not always increase during KMI, as vividness imagery is performed when relevant numerical information is provided.

Therefore, as described above, factors underlying the conflicting effects of MI (inhibit/improve) have not yet been clarified, and the clinical application of MI is still controversial. Based on the aforementioned background, we considered it important to compare the effects of the best MI strategies. We aimed to test the hypothesis that KMI with number visualization modulates motor skills without altering the excitability of the spinal motor neurons. Therefore, we analyzed the F-wave during MI, and change in motor skill after each MI strategy, in a healthy participant.

## Results

The speed of a skill under conditions of task precision is important for work and task performance. The performance indicators are as follows. Using the timings required to complete the pinch force adjustment (X) and initiate pinch force (Y), the time required to adjust to target MVC (s) was calculated for “X–Y”. In addition, the coefficient of variation (standard deviation / mean) was used as the index of misalignment of pinch force trajectory. The error value for the pinch force measurement was pinch force (kgf) actually exerted by the participant minus the pinch force (kgf) at 50% MVC determined for each participant. For example, if the target value (50% MVC) to be adjusted by the subject is 10 kgf, that value is taught in advance. While checking the pinch meter, the subject exerts 10 kgf, but if the adjustment does not go smoothly and the subject exerts a pinch force of 10.4 kgf, the error is 0.4 kgf. Since there are individual differences in pinch force for 50% MVC, it must be expressed as a ratio, which in the example is 4% error (error [%] = [exerting pinch force − 50% MVC] / 50% MVC × 100)^27^. This index was not a performance study item, but rather a precision item for skill assurance and was used to classify the recruited participants. However, none of the participants had more than 5% error from the 50% maximal voluntary contraction (MVC) in terms of pinch force error, and all participants were able to perform MI. The details along with the difficulty level of MI are shown in Table 1.

**Table 1:** Imagination sensitivity and participants’ reflections, and details of error of less than 5%.

Subject	Laterality quotient of Edinburgh Handedness Inventory	"Thumb to Finger tips " from KVIQ at 5Kd	KMI				Number visualizing				KMI with Number visualizing			
			Participants' reflections		Error (%)		Participants' reflections		Error (%)		Participants' reflections		Error (%)	
			Perform the MI	Difficulty of MI	Pinch task 1	Pinch task 2	Perform the MI	Difficulty of MI	Pinch task 1	Pinch task 2	Perform the MI	Difficulty of MI	Pinch task 1	Pinch task 2
1	100	4/5	Possible	Difficult	0.09764	1.33056	Possible	Difficult	− 1.2375	1.9922917	Possible	Easy	3.6210909	3.0384545
2	100	4/5	Possible	Difficult	3.9751765	− 1.8231765	Possible	Difficult	2.0813125	0.612625	Possible	Easy	− 0.0930625	− 0.4745625
3	100	4/5	Possible	Difficult	3.302	1.3183333	Possible	Difficult	3.2511667	4.0140833	Possible	Easy	2.6916667	2.5136667
4	100	5/5	Possible	Easy	1.6348148	− 3.4288148	Possible	Easy	0.87888	0.58592	Possible	Easy	− 2.9999	0.0366
5	80	5/5	Possible	Easy	− 0.3165	− 0.2729286	Possible	Easy	1.1703939	0.4675758	Possible	Easy	0.7666923	1.0836154
6	100	5/5	Possible	Easy	1.1710345	0.5606897	Possible	Easy	4.0039333	0.3418	Possible	Easy	− 1.4404	− 2.0752
7	90	4/5	Possible	Difficult	2.2644	1.8524	Possible	Difficult	− 3.4220667	− 3.3406667	Possible	Easy	0.803375	1.127625
8	100	4/5	Possible	Difficult	2.1152222	4.3362222	Possible	Difficult	− 1.30615	− 1.0315	Possible	Difficult	0.69275	3.51565
9	100	5/5	Possible	Easy	3.8615	4.6753333	Possible	Easy	0.769	1.5626	Possible	Easy	− 1.5445238	1.4345238
10	80	5/5	Possible	Easy	4.248	3.363	Possible	Easy	− 2.5399286	− 0.6652857	Possible	Easy	− 0.2207826	− 0.8576522
11	100	4/5	Possible	Difficult	1.6367826	0.7876522	Possible	Difficult	0.174	4.14125	Possible	Easy	0.075037	0.2559259
12	100	5/5	Possible	Easy	4.8278571	1.5363571	Possible	Easy	− 2.0783478	1.1856522	Possible	Easy	− 2.2059355	0.9836774
13	90	4/5	Possible	Difficult	− 0.6958	0.12815	Possible	Difficult	4.852	− 4.9778947	Possible	Easy	− 1.780359	1.1462051
14	100	4/5	Possible	Difficult	− 0.0687879	− 0.9195758	Possible	Difficult	4.4464375	4.4845625	Possible	Easy	− 0.3331765	3.7058824
15	100	4/5	Possible	Difficult	2.7248261	3.3484348	Possible	Difficult	2.98155	3.10365	Possible	Difficult	0.4842	− 2.5065
16	100	4/5	Possible	Difficult	0.2502	4.3396	Possible	Difficult	− 4.2053	1.8982	Possible	Easy	− 1.001	3.4851
17	100	4/5	Possible	Difficult	4.9001579	3.4706316	Possible	Easy	3.824	− 2.9058947	Possible	Easy	3.9203684	3.0048421
18	100	4/5	Possible	Difficult	2.6408	2.9866667	Possible	Difficult	1.6032	0.8300667	Possible	Difficult	1.3387333	− 0.1872
19	100	4/5	Possible	Difficult	1.69985	− 0.0244	Possible	Easy	0.0519	− 0.0702	Possible	Easy	0.0519	0.46385
20	100	4/5	Possible	Difficult	− 1.33665	− 0.2075	Possible	Easy	1.98975	1.95925	Possible	Easy	0.66225	2.4933
21	90	4/5	Possible	Difficult	4.7716842	4.8037895	Possible	Difficult	2.6033158	2.6836316	Possible	Easy	1.9447895	− 1.1551579

**Figure 1:**
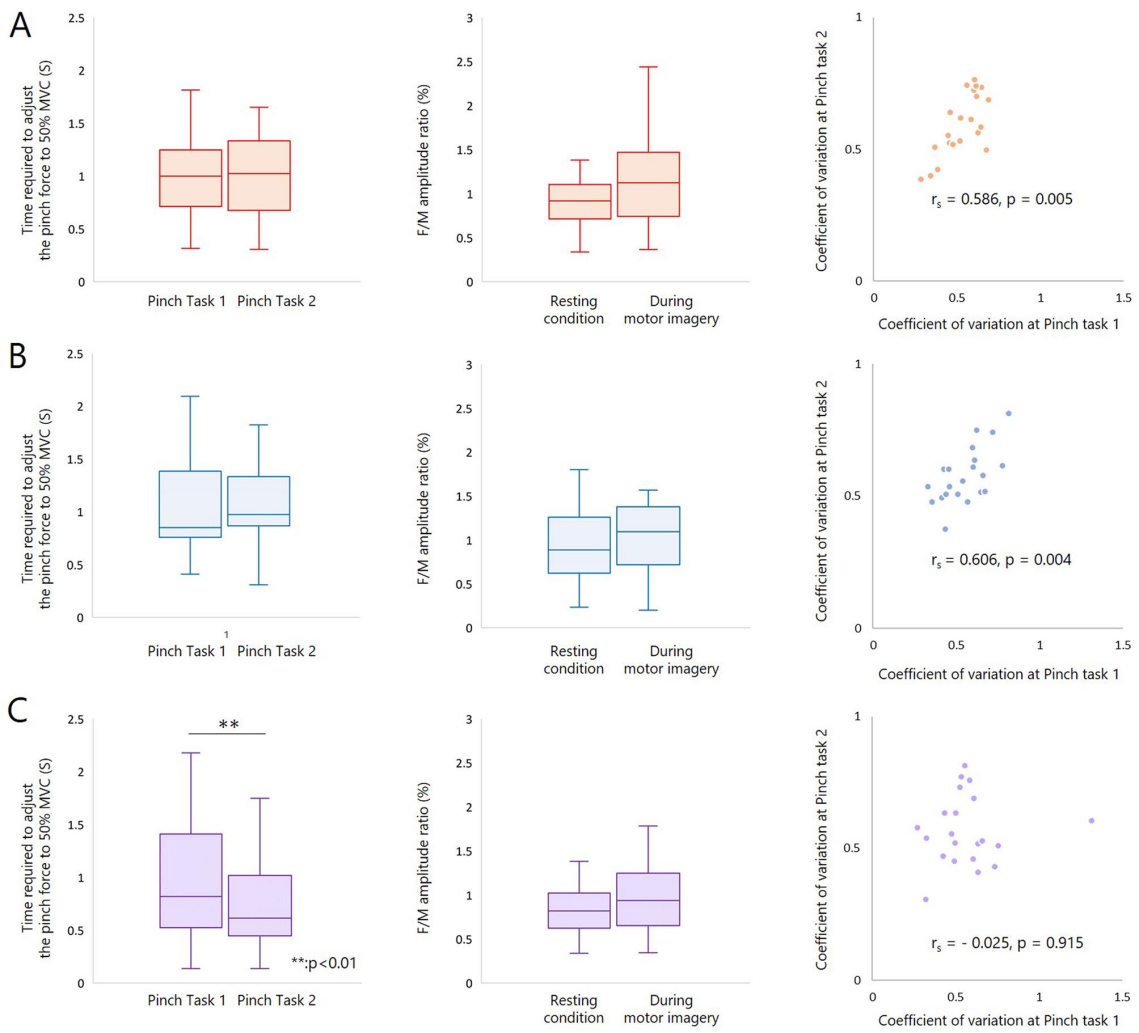
Changes in motor skills and F-waves with each motor imagery strategy. (**A**) In the KMI condition, there were no significant differences in the time required to adjust the pinch force to 50% MVC between each pinch task (r = 0.06, *p* = 0.768). There were no significant differences in the F/M amplitude ratio between the resting and KMI conditions (r = 0.30, *p* = 0.170). In the KMI condition, positive correlations were found between the coefficient of variation for each pinch task (r_s_ = 0.586, *p* = 0.005). (**B**) In the number visualizing condition, there were no significant differences in the time required to adjust the pinch force to 50% MVC between each pinch task (r = 0.04, *p* = 0.848). There were no significant differences in the F/M amplitude ratio between the resting and number visualizing conditions (r = 0.32, *p* = 0.140). In the number visualizing conditions, positive correlations were found between the coefficient of variation for each pinch task (r_s_ = 0.606, *p* = 0.004). (**C**) The time required to adjust the pinch force to 50% MVC was significantly shorter for the pinch task 2 than pinch task 1 in KMI with number visualizing condition (r = 0.69, *p* = 0.002). There were no significant differences in the F/M amplitude ratio between resting and KMI with number visualizing conditions (r = 0.28, *p* = 0.205). No correlations were observed between the coefficient of variation for each task in KMI with number visualizing condition (r_s_ =  − 0.025, *p* = 0.915).

**Figure 2:**
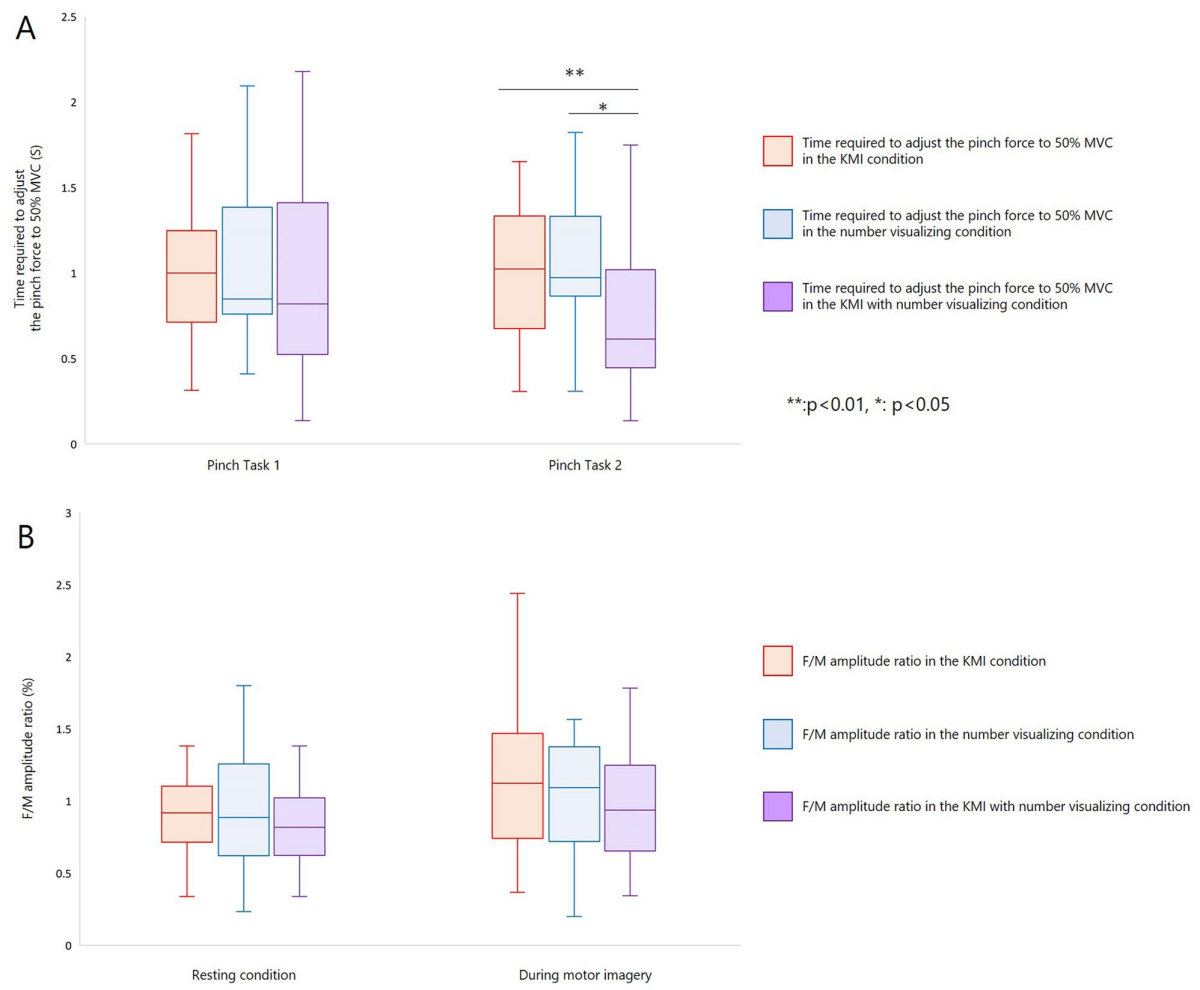
Comparison between each motor imagery strategy. (A) There were no differences in the time required to adjust the pinch force to 50% of maximum voluntary contraction (MVC) in pinch task 1 between the KMI, number visualizing, and KMI with number visualizing (Friedman’s test: DF = 2, *p* = 0.717). This confirmed that participants were able to properly perform the task under the relevant condition without being affected by the previous condition. There was a difference in the time required to adjust the pinch force to 50% MVC in pinch task 2 among the KMI, number visualizing, and KMI with number visualizing (Friedman’s test: DF = 2, *p* = 0.001). Moreover, the time required to adjust the pinch force to 50% MVC was shorter in the KMI with number visualizing condition than in the KMI condition (Wilcoxon signed-rank test: r = 0.73, *p* = 0.003). The time required to adjust the pinch force to 50% MVC was shorter for the KMI with number visualizing than in the number visualizing condition (r = 0.57, *p* = 0.030). (**B**) There were no differences in the F/M amplitude ratios between each MI condition at rest (Friedman’s test: DF = 2, *p* = 0.842) and during MI (Friedman’s test: DF = 2, *p* = 0.264).

The time required to adjust the pinch force to 50% MVC was 1.02 ± 0.45 s for KMI, 1.04 ± 0.49 s for number visualizing, and 1.00 ± 0.57 s for combined KMI with number visualizing condition in pinch task 1. In pinch task 2, the time required to adjust the pinch force to 50% MVC was 1.07 ± 0.56 s for KMI, 1.05 ± 0.40 s for number visualizing, and 0.77 ± 0.48 s for KMI with number visualizing. In KMI and number visualizing condition, there were no significant differences in the time required to adjust the pinch force to 50% MVC between each pinch task (KMI: r = 0.06, *p* = 0.768; number visualizing: r = 0.04, *p* = 0.848) (Fig. 1A,B). The time required to adjust the pinch force to 50% MVC was significantly shorter for pinch task 2 than pinch task 1 in KMI with number visualizing condition (r = 0.69, *p* = 0.002) (Fig. 1C). There were no significant differences in the time required to adjust the pinch force to 50% MVC in pinch task 1 for the KMI, number visualizing, and KMI with number visualizing condition (DF = 2, *p* = 0.717) (Fig. 2A). In pinch task 2, there was a difference in the time required to adjust the pinch force to 50% MVC among KMI, number visualizing, and KMI with number visualizing conditions (DF = 2, *p* = 0.001) (Fig. 2A). In order to further investigate these differences, multiple comparisons with Bonferroni’s correction were performed. The time required to adjust the pinch force to 50% MVC in pinch task 2 was significantly shorter for KMI with number visualizing condition than for the KMI condition (r = 0.73, *p* = 0.003) (Fig. 2A). Moreover, the time required to adjust the pinch force to 50% MVC in pinch task 2 was significantly shorter for the KMI with number visualizing condition than in the number visualizing condition (r = 0.57, *p* = 0.030) (Fig. 2A). However, in pinch task 2, there was no significant difference in the time required to adjust the pinch force to 50% MVC between the KMI and number visualizing conditions (r = 0.02, *p* = 2.793) (Fig. 2A). In the KMI and number visualizing conditions, positive correlations were found between the coefficient of variation for each pinch task at (KMI: r = 0.586, *p* = 0.005; number visualizing: r = 0.606, *p* = 0.004). No correlations were observed between the coefficient of variation for each task in KMI with number visualizing condition (r = −0.025, *p* = 0.915) (Fig.1A–C).

The F/M amplitude ratio reflects the number of backfiring anterior horn cells and the excitability of individual anterior horn cells. In other words, the F/M amplitude ratio reflects the excitability of lower motor neurons within the spinal cord. The F/M amplitude ratio was 0.98 ± 0.54% for KMI, 1.04 ± 0.73% for number visualizing, and 0.96 ± 0.62% for KMI with number visualizing condition in the resting state. For all MI conditions, the F/M amplitude ratio was 1.14 ± 0.50% for KMI, 1.10 ± 0.63% for number visualizing, and 0.96 ± 0.36% for KMI with number visualizing condition. There were not significant differences in the F/M amplitude ratio between the resting and MI conditions (KMI: r = 0.30, *p* = 0.170; number visualizing: r = 0.32, *p* = 0.140; KMI with number visualizing: r = 0.28, *p* = 0.205) (Fig. 1A–C). There were no significant differences in the F/M amplitude ratio in the rest periods among KMI, number visualizing, and KMI with number visualizing conditions (DF = 2, *p* = 0.842) (Fig. 2B). In addition, there were no significant differences in the F/M amplitude ratio during KMI, number visualizing, and KMI with number visualizing conditions (DF = 2, *p* = 0.264) (Fig. 2B).

## Discussion

Therefore, as described above, factors underlying the conflicting effects of MI (inhibit/improve) have not yet been clarified, and the optimal MI strategy is still controversial. In this study, we tested the hypothesis that KMI with number visualization modulates motor skills without altering the excitability of the spinal motor neurons. Therefore, we analyzed the excitability of spinal motor neurons (F/M amplitude ratio) during MI, and change in motor skill (the time required to adjust the pinch force to 50% MVC, and the coefficient of variation) after each MI strategy in healthy participants. The time required to adjust the pinch force to 50% MVC in pinch task 2 after MI was significantly shorter in the KMI with number visualizing condition than in the KMI condition. For KMI with number visualizing, significantly shorter time was required to adjust the pinch force to 50% MVC for the pinch task 2 than pinch task 1. However, there were no significant differences in the time required to adjust the pinch force to 50% MVC between the pinch tasks in KMI condition. The participants reported that they could perform MI; however, they found it difficult to perform KMI. KMI relies on the feeling of muscle movement, which can usually be achieved after training^16^. Additionally, KMI is more difficult to perform than VMI^29^. Therefore, participants who have difficulty in performing KMI automatically transition to VMI^30^. There is a strong association between KMI and motor performance outcome^13^, and the error rate of precision right finger tapping decreased after KMI. However, VMI was negatively associated with performance^14^. Therefore, it is possible that in the KMI condition, participants did not improve their motor skills because they only performed VMI. Request for participants to "imagine the pinch force increasing numerically on the pinch meter display" may lead to greater kinesthetic involvement than VMI. The imagery property of mentally increasing numbers has properties more similar to KMI than VMI^31^. The participants in this study reported that the KMI with number visualizing was easier than KMI in terms of difficulty of MI. Therefore, the use of numerical information may help in performing KMI correctly, even in participants who find it difficult to perform KMI without this information. However, it is important to provide numerical information that is relevant to the MI because irrelevant numerical information may not be useful^19^. In the KMI with number visualizing condition, not only was the time to adjust the pinch force to 50% MVC reduced, but the coefficient of variation was also not correlated. When MI changes the motor strategy, the coefficient of variation changes with each pinch task. This is because the standard deviation and the average value of the pinching force are different. In other words, in the KMI with number visualizing condition, the time to adjust the pinch force to 50% MVC may be shortened due to the change in motor strategy. MI that seeks to acquire new motor skills can improve motor skills^32^ and induce central nervous system adaptation^33^. These results suggest that performing KMI correctly improves motor performance.

The present study showed there was no significant difference in the F/M amplitude ratio between KMI, number visualizing, and KMI with number visualizing conditions in the resting and different MI conditions. There are two hypotheses regarding lack of increase in the excitability of spinal motor neurons with MI. First, MI is difficult to implement. Excitability of spinal motor neurons does not increase in case of difficulty in performing MI^34^. If there is great difficulty in performing MI, motor skills do not improve^35^. Second, the increase in excessive excitability of spinal motor neurons was suppressed because of the vivid MI^36^. Participants who improved their precision pinch force control skills by performing MI performed vivid MI, which suppressed the increase in excessive excitability of spinal motor neurons^28^. The excitability of spinal motor neurons may not increase during MI due to conflicting various, and two possible patterns may emerge: the imagery may not be smooth or it may be vivid. In the KMI and number visualization conditions, participants were able to perform motor imagery, albeit with a high degree of difficulty. Furthermore, no improvement in the time required to adjust the pinch force to 50% MVC was observed in these conditions, suggesting that a lack of change in F/M amplitude ratio was largely due to the first hypothesis. Additionally, the KMI with number visualization condition was relatively less difficult to implement, suggesting that vivid MI (second hypothesis) may have been performed, because the time required to adjust the pinch force to 50% MVC improved after the KMI with number visualizing.

In conclusion, this study evaluated the change in motor skill in terms of changes in the spinal motor neuron excitability using MI strategies. KMI relies on the sensation of moving muscles and is a highly challenging MI. However, given that the imagery property of mentally increasing numbers is more similar to KMI than to VMI, providing numerical information related to MI can enable the execution of KMI. The KMI condition with number visualization suggested the possibility of performing vivid MI with relatively low difficulty. Conflicting results have been reported regarding spinal motor neuron excitability during MI. The spinal motor neuron excitability does not increase if, for example, the imagery is difficult to perform or, conversely, if it is performed very vividly. In this study, it is likely that KMI did not increase spinal motor neuron excitability because KMI was difficult to perform. However, in the KMI condition with number visualization, spinal motor neuron excitability did not increase because MI was relatively less challenging than vivid MI based on the participants' reflections of introspection. Thus, the KMI condition with numerical visualization seems to accomplish vivid imagery, reduce the time to adjust the pinch force to 50% MVC, and even change the motor strategy. Since differences in only MI strategy affected the results under the same conditions, this study may provide an example of an effective strategy. Future studies should examine whether or not the accompanying number visualization is effective during different motor tasks.

## Materials and methods

### Participants

Before conducting the experiments, the appropriate sample size was estimated by power analysis using the software G∗power (version 3.1.9.4)^37^ for paired t-test, Wilcoxon signed-rank test (matched pairs), and analysis of variance (ANOVA; repeated measure and within factors). The paired t-test was used when the data were normally distributed, and Wilcoxon signed-rank test was used when data were not normally distributed. The type of power analysis was set to “a priori: compute required sample size given α, power, and effect size”, “difference between two dependent means (matched pairs)”, and “Wilcoxon signed-rank test (matched pairs)”; the effect size dz was set to 0.8, α error probability was set to 0.05, and power (1−β error probability) was set to 0.8. The sample size was calculated to be 15. For ANOVA for repeated measure and within factors, the effect size f was set to 0.4, α error probability to 0.05, power (1−β error probability) to 0.8, number of groups to 1, number of measurements to 3, correlation among repetitive measures to 0.5, and non-sphericity correction epsilon to 1. The sample size was calculated to be 12. The sample size estimation considered the possibility of high effect sizes in this study because our previous studies^20,38^ showed a high effect size; therefore, an appropriate sample size was necessary to prevent error. Therefore, 21 healthy participants (13 men and 8 women; mean age: 24.8 ± 5.5 years) were included to ensure that the actual sample size was greater than the calculated sample size. We did not exclude any participants due to an error of more than 5% (error [%] = [exerting pinch force – 50% MVC] / 50% MVC × 100) from the 50% MVC to examine the time required for accurate adjustment^27^. The participants were determined to be right-handed using the Edinburgh Handedness Inventory to control the condition of hand dominance (Table 1). Therefore, the left hand, i.e., the non-dominant hand side of participants, were selected as the side to perform the pinch action. All participants provided informed consent prior to the commencement of the study, and all participants consented to the publication of their data. The experiments were conducted in accordance with the Declaration of Helsinki. All methods were performed in accordance with the relevant guidelines and regulations. The study was approved by the Research Ethics Committee at Kansai University of Health Sciences (approval number: 19-37). Participants with pacemakers, cardiac catheters, or venous pressure measurement leads inserted directly into the heart were excluded. In addition, pregnant individuals were excluded.

### Study procedure

The motor task assessed in the present study was pinch force control time because the speed under conditions of task precision of a skill are essential for work and task performance^39^. The MVC of pinch force was calculated prior to practicing the pinch task using a digital pinch meter. In addition, the participants were informed of the 50% MVC in kgf by observing the display of the digital pinch meter (F340A; Unipulse, Inc., Tokyo, Japan), and it was explained that this was the target value.

**Figure 3:**
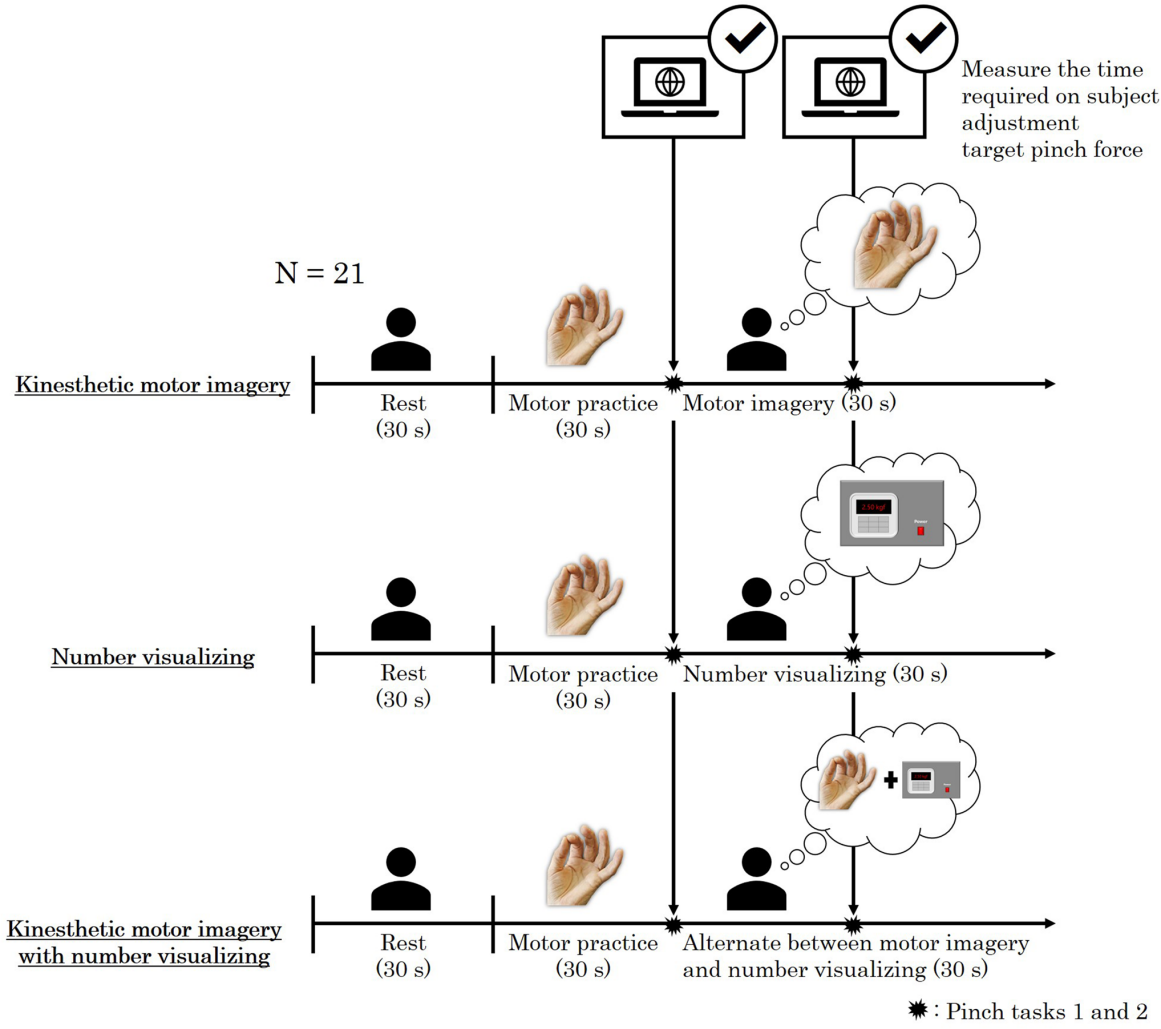
Study procedure. We examined the F-waves of the left thenar muscles after stimulating the left median nerve at the wrist at rest and during MI trials performed in a random order. The KMI condition consisted of imagining performing muscle contractions; the number visualizing condition consisted of imagining the pinch force increasing numerically; and KMI with number visualizing condition consisted of alternating between KMI and number visualization. Before and after MI, the ability of the participants to quickly adjust to the target pinch force was evaluated, and the time required to adjust to the target was compared between the conditions.

In the resting condition, the participants were asked to lie comfortably in the supine position for 30 s and electro-stimulation was performed to record F-waves. Then, they were asked to practice the pinching movement repeatedly for 30 s while quickly adjusting to 50% MVC by referring to the display of the digital pinch meter. For the practice session, 6 sequences were set up with an interval of 5 s during which the participants had to adjust to 50% MVC within a maximum of 5 s. After adjustment for 50% MVC, the participants had to decompress and prepare for the next sequence by performing 6 repetitions in 30 s, which was enough practice^6^. In pinch task 1, the participants were instructed to adjust to 50% MVC as quickly as possible while observing the display. Six sequences were provided in the practice session. However, during the pinch task, the motor skill was evaluated by performing the task only once. The participants were instructed to push only one button to finish the adjustment to 50% MVC. The button was connected to the measurement PC, and a spike appeared on the PC screen when the button was pushed. Spikes were used to analyze the timing as a measure of the motor skills. Next, the participants were asked to perform one of the three MI conditions in conjunction with electro-stimulation and F-wave recording. MI was performed similar to the motor practice, while imagining repeated execution of a pinching action. The KMI condition involved asking the participants to “imagine performing muscle contractions for the pinch action”, the number visualizing condition involved asking the participants to “imagine the pinch force increasing numerically on the pinch meter display”, and the KMI with number visualizing condition involved asking the participants to “alternate between imagining muscle contractions with the pinch action and the pinch force increasing numerically on the pinch meter display”^8^. Finally, for pinch task 2, the participants were asked to repeat pinch task 1. Although no actual movement was involved during the imagery task, physical exercises were performed before each imagery task was performed. In other words, in this study, imagery and physical exercises were combined in a certain order to maximize the correct imagery. Motor performance reportedly improves by repeated performance of MI^40^; therefore, the three different MI conditions were randomly performed on 3 separate days (Fig. 3). A washout period of at least one week was provided on each condition implementation day to ensure that each participant could properly perform the condition without being affected by the previous condition.

**Figure 4:**
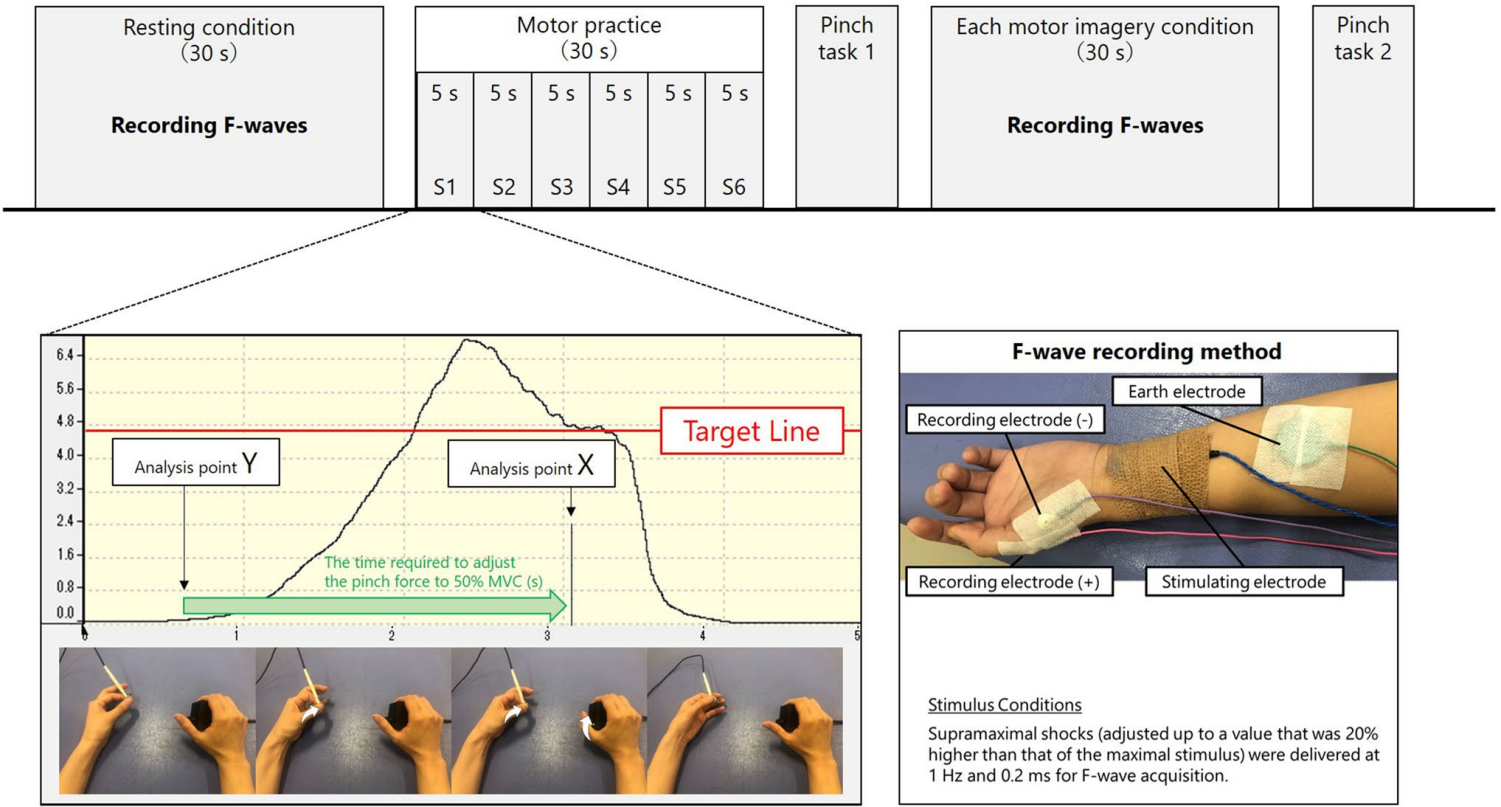
Exercise task and F wave measurement conditions. Within each pinch task, the participants were instructed to push a button while subjectively thinking of adjusting to 50% MVC. The button was connected to a measurement PC, and spikes appeared on the measurement PC screen when the button was pushed. For the practice, 6 sequences (S1 − 6) with 5 s intervals were set up in which participants had to adjust to 50% MVC within a maximum of 5 s. A Viking Quest Electromyography machine (Natus Medical, Inc., Tokyo, Japan) was used to record F-waves at rest and in each motor imagery condition.

A recording software (Vital Recorder2; Kissei Comtec, Inc., Nagano, Japan) and a versatile biological analysis system (Bimutas-Video; Kissei Comtec, Inc.) were used to analyze pinch tasks in each MI condition. The assessment index included the error from 50% MVC, the time required to adjust the pinch force to 50% MVC, and the coefficient of variation. The error value of the measured pinch force from 50% MVC was recorded at the time of spike appearance. The error value for the pinch force measurement was pinch force (kgf) exerted by the participant minus the pinch force (kgf) at 50% MVC determined for each participant. For example, if the target value (50% MVC) to be adjusted by the subject is 10 kgf, that value was taught in advance. While checking the pinch meter, the subject exerts 10 kgf, but if the adjustment does not go smoothly and the subject exerts a pinch force of 10.4 kgf, the error is 0.4 kgf. Since there are individual differences in pinch force for 50% MVC, it was expressed as a ratio, which in this example is 4% error (error [%] = [exerting pinch force – 50% MVC] / 50% MVC × 100)^27^. However, this index was not a performance study item, but rather a precision item for skill assurance and was used to classify the recruited participants. The performance indicators are as follows. Using the timing of spike appearance X and initiation of pinch force Y, the time required to adjust to target MVC (s) was calculated for “X − Y” (Fig. 4). If MI modulates the motor strategy (overshoot or undershoot causing oscillations above and below the target), the standard deviation and mean of the pinch force should change. Therefore, the coefficient of variation (standard deviation / mean) was defined as the index of misalignment of pinch force trajectory.

A Viking Quest Electromyography machine (Natus Medical, Inc., Tokyo, Japan) was used to record F-waves in the resting condition and each MI condition. Supramaximal shocks (adjusted to a value 20% higher than that of the maximal stimulus) were applied to the left median nerve at the wrist. Electrical stimulation was used to induce the F wave. Electrical stimulation during MI does not interfere with the imagery task^22^. F-wave amplitude is not constant; however, F-wave amplitude based on the maximum M-wave amplitude generates constant values. Therefore, supramaximal shocks were used to record the maximum M-waves. The stimulus was delivered 30 times at 1.0 Hz for 0.2 ms. F-waves were recorded from the left thenar muscles using a pair of disks that were attached to the skin over the belly of the thumb and the bones of the metacarpophalangeal joint of the thumb using collodion. The stimulating electrodes comprised of a cathode placed over the left median nerve 3 cm proximal to the palmar crease of the wrist joint and an anode placed 2 cm proximally.

Imagination sensitivity is crucial since differences between participants strongly influence the task measures^41^. "5Kd Thumb to Fingertip" was extracted using the Kinesthetic and Visual Imagery Questionnaire (KVIQ) in Japanese version, a questionnaire that assesses the ability to recall MI^42^. The participants were asked whether they were able to perform MI (possible or impossible) and the difficulty thereof (difficult or easy).

### Data analysis

The independent variable in this study was the MI strategy, whereas the time required to adjust the pinch force to 50% MVC, the coefficient of variation, and the F/M amplitude ratio were the dependent variables. The time required to adjust the clamping force to 50% MVC, the coefficient of variation, and the F/M amplitude ratio were continuous variables. We discarded the outlier data with mean ± 5 times of standard deviation (SD). The Shapiro-Wilk tests revealed that the data were not normally distributed.

The three experimental conditions (KMI, number visualizing, and combined KMI and number visualizing) and the time required to adjust the pinch force to 50% MVC before and after exercise imagery were compared using Wilcoxon's signed-ranked test. The F/M amplitude ratios were compared at rest and during MI. Moreover, the relationships between the coefficient of variation for each pinch task were examined using Spearman’s correlation coefficients.

The time required to adjust the pinch force to 50% MVC in pinch tasks 1 and 2, and the F/M amplitude ratios for each MI strategy in the resting condition were compared across conditions using the Friedman’s test. If necessary, a Wilcoxon signed-rank test with Bonferroni’s correction was used for multiple comparisons.

The effect size (r) was calculated using the Wilcoxon signed-rank test. Statistical significance was set at *p* < 0.05. We used IBM SPSS software (version 26.0; IBM Corp., Armonk, NY, USA) for the statistical analyses.

## Data Availability

The datasets generated during and/or analyzed during the current study are available from the corresponding author on reasonable request.
